# Evaluation of Autoregressive Models for Predicting Two‐Dimensional Mandibular Landmark Displacement During Pubertal Growth

**DOI:** 10.1111/ocr.70150

**Published:** 2026-05-26

**Authors:** Abdullah Al Fahad, Jared Brown, Ali Walid Elhag, Azeezjamal Samad Lakhani, George Eckert, Marwa Baraka, Mohammad Al Hasan, Hakan Turkkahraman

**Affiliations:** ^1^ Department of Computer Science, Luddy School of Informatics, Computing and Engineering Indiana University Indianapolis Indiana USA; ^2^ Department of Orthodontics and Oral Facial Genetics Indiana University School of Dentistry Indianapolis Indiana USA; ^3^ Indiana University School of Dentistry Indianapolis Indiana USA; ^4^ Department of Biostatistics and Health Data Science Indiana University, School of Medicine Indianapolis Indiana USA; ^5^ Department of Pediatric Dentistry and Dental Public Health Alexandria University School of Dentistry Alexandria Egypt

**Keywords:** chin, growth and development, mandibular condyle, prediction algorithms

## Abstract

**Objectives:**

To predict mandibular landmark displacement during pubertal growth using autoregressive models.

**Materials and Methods:**

The study included 225 subjects with complete cephalometric radiographs from ages 10 to 18. Data were split by subject into training (180; 83 males, 97 females) and test sets (45; 21 males, 24 females). Mandibular landmarks analysed were Condylion, Gonion, Menton, Pogonion, and Point B. Radiographs were aligned using the Sella–Nasion line with Sella as the origin, and 2D coordinates for each landmark were recorded over time. An autoregressive model predicted future landmark positions. Accuracy was measured using mean absolute error and the percentage of predictions within 2 mm of true values. Logistic regression with generalised estimating equations evaluated the effects of coordinate dimension, landmark type, prediction interval (1–4 years), prediction age (11–18), and sex.

**Results:**

Females demonstrated higher predictive accuracy than males (65% vs. 49%), by using the percentage of predictions within 2 mm of the true coordinates. Accuracy declined with longer prediction intervals: 66% (1‐year), 58% (2‐year), and 46% (4‐year). Condylion was the most predictable landmark (71%), followed by Point B (63%) and Gonion (61%), whereas Menton was least predictable (40%). Accuracy was similar between *x*‐ and *y*‐dimensions (58% vs. 56%). Overall predictive accuracy decreased substantially when Euclidean distance was used instead of separate coordinate‐based assessments, ranging from 39% for Condylion to 18% for Menton.

**Conclusions:**

Autoregressive models can generate clinically meaningful short‐term predictions of mandibular growth, particularly for selected landmarks and among female subjects. However, predictive performance declines over longer time intervals and varies substantially across anatomical sites, with Condylion demonstrating the highest accuracy and Menton showing the greatest variability.

## 1 Introduction

Although the number of adults seeking orthodontic treatment has increased in recent years [1], the majority of orthodontic patients remain growing children and adolescents [2]. Growth introduces an additional layer of complexity to an already challenging treatment decision‐making process, as the craniofacial complex that supports the dentition undergoes continuous structural change during development [3]. In patients with skeletal Class II and Class III discrepancies in particular, accurate prediction of craniofacial growth is critical, since future growth may either enhance treatment outcomes [4] or compromise both treatment success and long‐term stability [5].

Among the components of the craniofacial complex, the mandible exhibits the greatest postnatal growth potential. Its size and morphology are largely influenced by genetic determinants [6, 7]; however, because it articulates with the cranial base via the temporomandibular joint and is functionally integrated with surrounding structures, its growth trajectory is also influenced by adjacent muscular, skeletal and dental units—particularly the maxilla and maxillary dentition. Environmental and functional factors, including mode of respiration [8] and tongue posture [9] have also been proposed to influence mandibular growth patterns. While the evidence supporting environmental modulation is less robust than that supporting genetic control, mandibular growth is widely accepted as multifactorial and highly variable among individuals.

Despite its clear clinical importance, accurate prediction of pubertal craniofacial growth remains one of the most persistent challenges in orthodontics. Growth variability in timing, magnitude, velocity, and direction contributes substantially to this difficulty [10]. The pubertal growth spurt is particularly complex, characterised by nonlinear acceleration and deceleration phases that differ between individuals and between sexes [11, 12]. Decades of research have sought to develop reliable predictive models [13, 14, 15]; however, a universally accepted and generalisable framework has yet to emerge.

Recent studies applying machine learning techniques to longitudinal craniofacial growth data have provided important insights [16, 17, 18, 19]. Analyses predicting post‐pubertal mandibular length and growth direction (e.g., *Y*‐axis of growth) demonstrated that the strongest predictors were often the same measurements obtained at earlier timepoints [16, 17, 18]. These findings suggest that prior size and spatial configuration encode substantial predictive information regarding future development. This observation highlights an important conceptual shift: rather than treating growth prediction as a static pre–post problem, it may be more appropriate to frame it as a time‐series forecasting task.

Cephalometric landmarks do not change independently or instantaneously; instead, their positions evolve sequentially over time, forming coordinated spatial trajectories. Viewing landmark displacement as a time series emphasises temporal dependency—where current anatomical states are statistically related to previous states—and allows modeling of growth as a dynamic process. However, explicit time‐series approaches remain underrepresented in craniofacial growth research. Most longitudinal statistical models in orthodontics have relied on mixed‐effects or Bayesian hierarchical frameworks designed primarily for scalar measurements or limited timepoint comparisons [20, 21, 22, 23], rather than for direct modeling of two‐dimensional coordinate trajectories.

Autoregressive (AR) models offer several unique advantages in this context. First, AR models are specifically designed to capture temporal dependency by modelling current observations as a function of previous states [24, 25]. This is particularly well suited to biological growth processes, where continuity and inertia characterise developmental change. Second, AR models are parsimonious yet powerful; they leverage the intrinsic information contained within an individual's prior trajectory without requiring large numbers of external predictors. In growth prediction, where inter‐individual variability is substantial but intra‐individual continuity is strong, this property is especially advantageous. Third, AR modelling naturally accommodates sequential clinical data collection [24]. Orthodontic records are typically obtained at multiple timepoints during growth, and AR frameworks can update predictions dynamically as new data become available. This enables iterative refinement of individualised growth forecasts rather than reliance on a single baseline assessment. Fourth, when applied to coordinate‐based landmark data, AR models preserve spatial information and allow prediction of both magnitude and direction of displacement [26, 27]. Modelling two‐dimensional trajectories directly avoids reduction of complex spatial change into isolated scalar measurements, thereby maintaining geometric fidelity [26, 27]. Finally, AR approaches provide a transparent and interpretable baseline for time‐series growth prediction [24, 25]. Unlike more complex ‘black‐box’ machine learning architectures, AR models allow explicit quantification of temporal influence (e.g., lag structure), facilitating biological interpretation and comparison across modelling strategies [24, 28]. Establishing such a baseline is essential before progressing to more computationally intensive methods.

In this study, we investigated whether AR models can predict pubertal displacement of mandibular landmarks within clinically acceptable error thresholds. Through this study, we aimed to clarify how model design influences accuracy and to establish a methodologically transparent foundation for future development of advanced, data‐driven approaches to individualised mandibular growth prediction.

## 2 Materials and Methods

### 2.1 Ethics

This study was reviewed by the Institutional Review Board (IRB) and determined to be not human subjects research (NHSR); therefore, no further IRB review was required.

### 2.2 Dataset and Preprocessing

All data were obtained from the American Association of Orthodontists Foundation (AAOF) Legacy Collection. Because our goal was to predict the next landmark position, subjects were included if they had consecutive lateral cephalometric radiographs taken between ages 10 and 18 years with complete landmark annotations; those with missing records or who had gone orthodontic treatment throughout the observation period were excluded. A total of 225 subjects met the inclusion criteria. After filtering, the analytic sample comprises 225 subjects with landmark coordinates. All data were collected under specialist supervision. The final cohort included 104 males and 121 females; by Angle classification, 92 subjects were Class I, 111 were Class II, and 22 were Class III.

All serial lateral cephalograms were previously pre‐registered and superimposed on the Sella–Nasion (SN) line, with Sella as the origin (0, 0). For each subject s and landmark ℓ, we extracted 2D Cartesian coordinates $p_{t}=\left(x_{t},y_{t}\right)$ at discrete time points t. Sequences with fewer than two consecutive time points for a given landmark were excluded. To ensure comparability, we further restricted ages to 10–18 years. We performed a subject‐level split to prevent identity leakage between the training and test sets. The final male cohort included 83 subjects in the training set and 21 in the test set. The female cohort included 97 subjects in the training set and 24 in the test set.

### 2.3 Problem Formulation

We modelled growth as first‐order autoregression [29] (AR (1)) at the landmark level. For each subject and landmark, the next 2D position $p_{t+1}=\left(x_{t+1},y_{t+1}\right)$ was predicted from the immediately preceding position $p_{t}=\left(x_{t},y_{t}\right)$

$$p_{t+1}=f_{\theta }\left(\phi \left(p_{t}\right)\right)+\varepsilon ,$$

where $f_{\theta }$ was a parametric regressor and $\phi \left(\cdot \right)$ was a feature map. We evaluated degree‐1 (linear) and degree‐2 (quadratic) polynomial features:

$$\begin{array}{c} \phi_{1}\left(p_{t}\right)=\left[x_{t},y_{t}\right],\\ \phi_{1}\left(p_{t}\right)=\left[x_{t},y_{t},x_{t}^{2},x_{t}y_{t},y_{t}^{2}\right]. \end{array}$$

### 2.4 Sex‐Adaptive Joint–xy Regressor

Building on the AR formulation, we implemented $f_{\theta }\left(\cdot \right)$ as a ridge‐regularised linear regressor that predicts the next landmark coordinates jointly. In practical terms, the model learns a stable, data‐driven rule for how each landmark tends to move from one time point to the next, while allowing this movement to differ by sex when the data support it.

For each landmark and time step, the model:

1. Takes the most recent 2D landmark position $p_{t}=\left(x_{t},y_{t}\right)$
2. Constructs features $\phi \left(p_{t}\right)$ using either degree‐1 (linear) or degree‐2 (quadratic) polynomials as defined previously; and
3. Outputs the predicted next position $\left({\hat{x}}_{\left(t+1\right)},{\hat{y}}_{\left(t+1\right)}\right)$.

### 2.5 Preprocessing

All input features were standardised using the training set mean and standard deviation. This step ensures that no single feature dominates simply because it has a larger numeric scale, and it makes the ridge regularisation behave consistently across features.

### 2.6 Sex‐Adaptive Design

The flowchart of the predictive model is shown in Figure 1. Rather than treating sex as a numeric input, we used sex only to select among fitted regressors. Specifically, we fit:

1. *Pooled regressor* trained on all subjects (female + male). This serves as the default and provides a robust estimate of overall growth trends.
2. *Female channel* trained only on female subjects (when enough data are available)
3. *Male channel* trained only on male subjects (when enough data are available).

**FIGURE 1:**
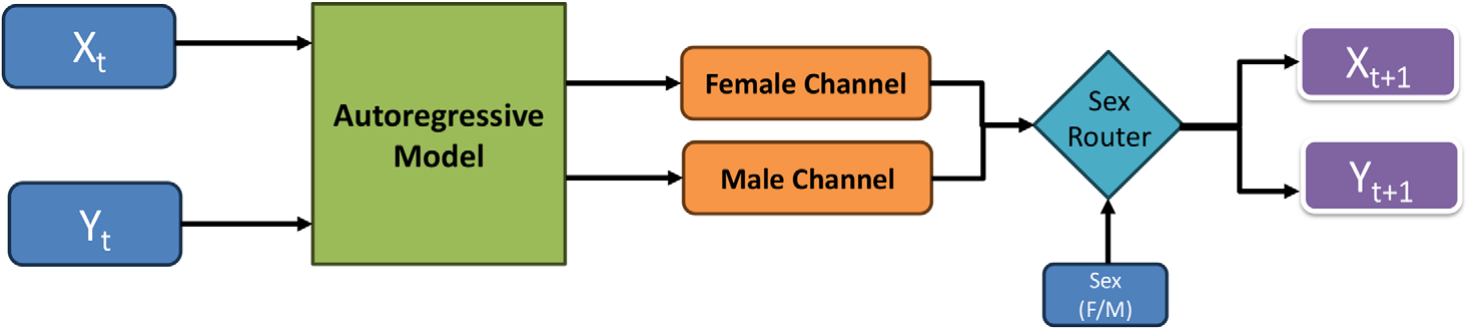
Flow‐chart diagram of the AR model.

For each test sample, when sex information was available, predictions were generated using the corresponding sex‐specific channel. If sex was missing or subgroup data were insufficient, the pooled regressor was used instead. This approach shares information across the entire cohort through the pooled model, while still allowing sex‐specific adjustments when supported by the data.

### 2.7 Model Selection and Evaluation

We selected the polynomial degree $\delta \in \left\{1,2\right\}$ and ridge regularisation constant α using subject‐grouped K‐fold cross‐validation on the training set, choosing the configuration that minimises validation mean absolute error (MAE) computed from 2D Euclidean landmark errors. After selecting $\left(\alpha ,\delta \right)$, we refitted the model on all training subjects and reported performance once on a held ‐out test set of subjects.

### 2.8 Statistical Analyses

Generalised estimating equation (GEE) methods applied to logistic regression were used to evaluate the effects of measurement dimension (x or y), landmark (Condylion, Gonion, Menton, Pogonion, Point B), prediction interval (1, 2, or 4 years), prediction age (11–18 years), and patient sex on the percentage of predicted measurements within 2 mm of the true measurement. A similar analysis was performed using Euclidean distance from the combined x and y dimensions. A two‐sided 5% significance level was used for all tests. Analyses were performed using SAS and R.

## 3 Results

Bland–Altman plots illustrating the distribution of differences between the predicted and actual x‐ and y‐coordinates of all mandibular landmarks are shown in Figures 2 and 3. The results of the GEE logistic regression analyses, conducted separately for the *x*‐ and *y*‐dimensions and for the combined Euclidean distance, are summarised in Table 1. Due to sparse data in some factor combinations, a full factorial model could not be fitted. Therefore, a reduced model including all two‐way interactions was initially examined, and non‐significant interactions were sequentially removed. Higher‐order interactions were also evaluated but were not statistically significant.

**FIGURE 2:**
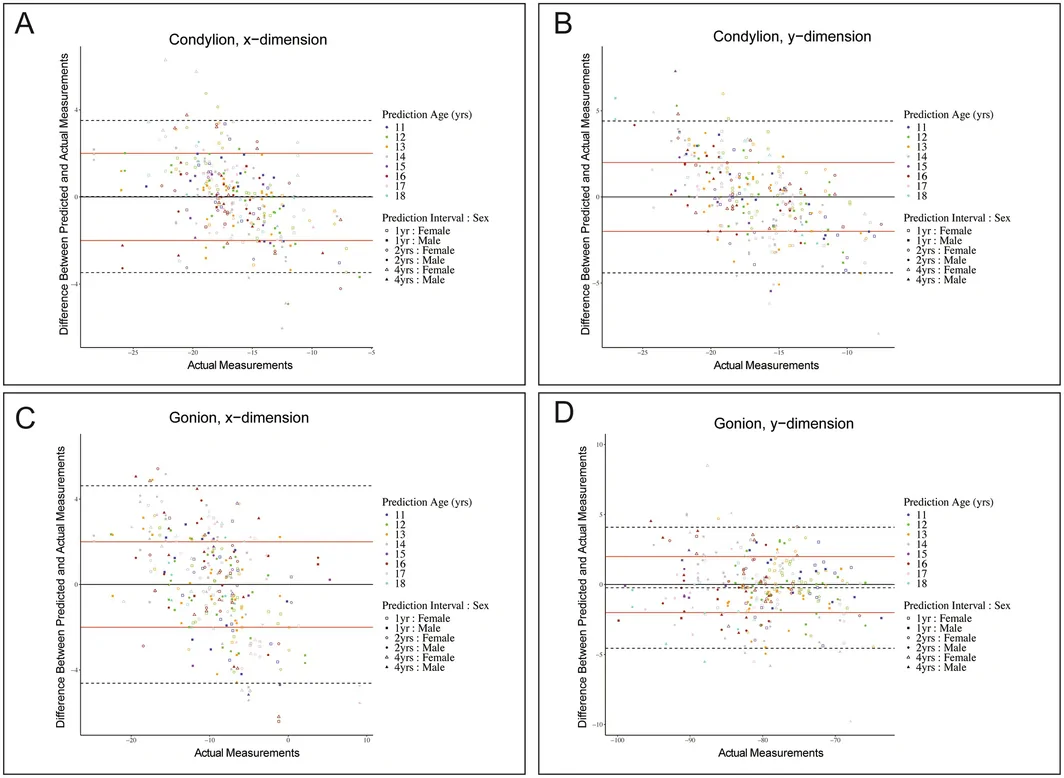
Bland–Altman plots illustrating the distribution of differences between the predicted and actual *x*‐ and *y*‐coordinates of (A, B) Condylion and (C, D) Gonion. Black dashed lines show the mean difference and mean difference ±2 standard deviations of the difference. Red lines indicated differences within ±2 mm.

**FIGURE 3:**
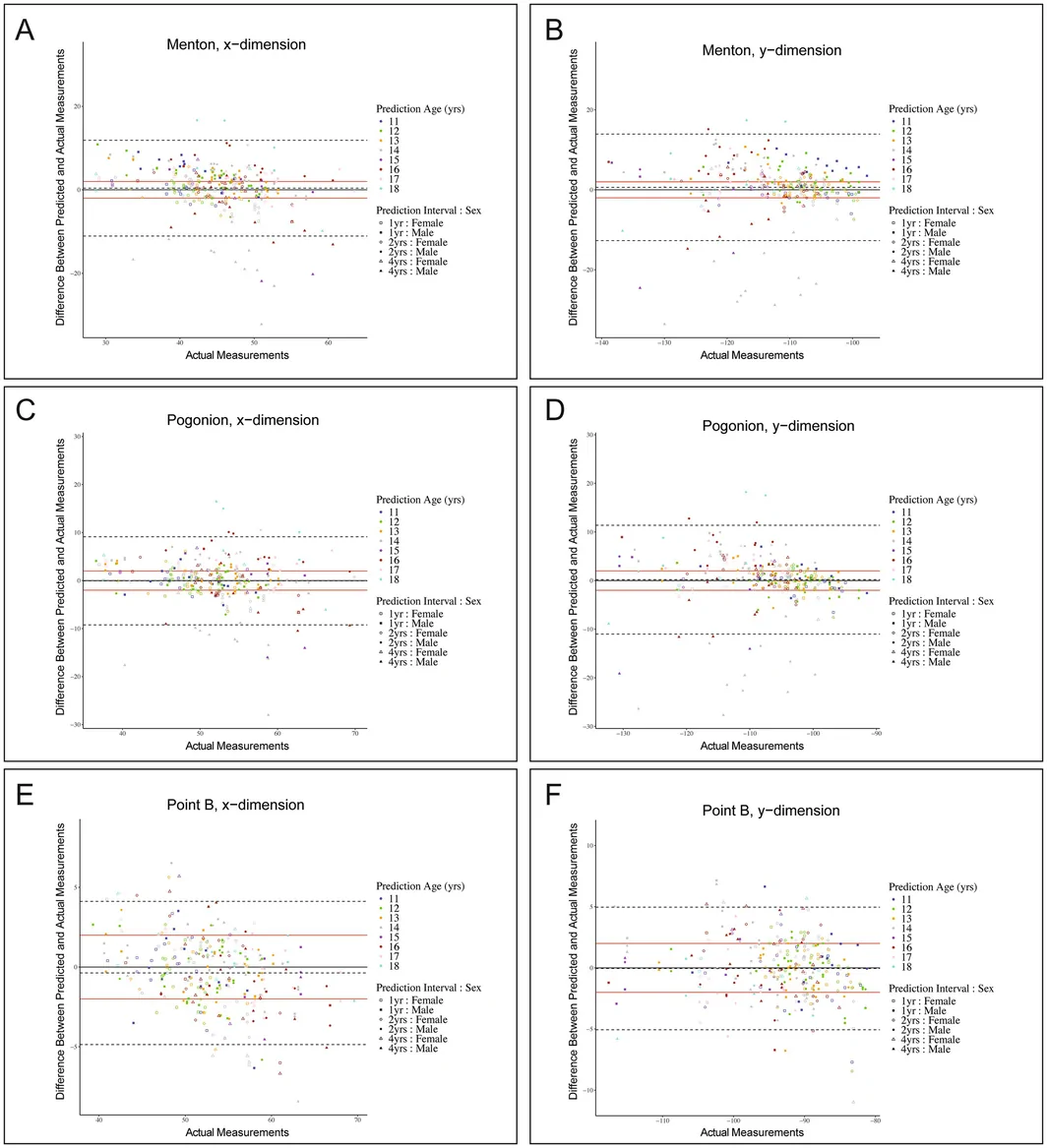
Bland–Altman plots illustrating the distribution of differences between the predicted and actual *x*‐ and *y*‐coordinates of (A, B) Menton, (C, D) Pogonion and (E, F) Point B. Black dashed lines show the mean difference and mean difference ±2 standard deviations of the difference. Red lines indicated differences within ±2 mm.

**TABLE 1:** Results of the generalised estimating equation (GEE) logistic regression analyses with *x* and *y* coordinates evaluated separately, and combined using Euclidian distance.

	Source	DF	Chi‐square value	*p*
*X* and *Y* coordinates	Dimension	1	1.22	0.270
	Landmark	4	19.04	0.001
	Prediction interval	2	13.90	0.001
	Prediction age	7	12.77	0.078
	Sex	1	12.56	0.000
	Dimension*Landmark	4	12.27	0.015
	Dimension*Prediction age	7	15.81	0.027
	Dimension*Sex	1	8.38	0.004
	Landmark*Prediction interval	8	18.73	0.016
	Landmark*Sex	4	24.54	0.000
Euclidean distances	Landmark	4	16.63	0.002
	Prediction interval	2	14.41	0.001
	Prediction age	7	17.00	0.017
	Sex	1	8.18	0.004
	Landmark*Sex	4	22.49	0.000

When the x and y dimensions were analysed separately, landmark type, prediction time interval, and sex demonstrated significant main effects on predictive accuracy (*p* < 0.05). In contrast, the main effects of dimension (x vs. y) and prediction age were not statistically significant (*p* > 0.05). All two‐way interactions were statistically significant (*p* < 0.05), with the interaction between landmark and sex showing the strongest effect (χ^2^ = 24.54, *p* < 0.001).

When predictive accuracy was evaluated using Euclidean distance, all factors—including landmark type, prediction time interval, prediction age, and sex—showed significant main effects (*p* < 0.05). A significant two‐way interaction between landmark and sex was also observed (*χ* ^2^ = 22.49, *p* < 0.001).

Model‐based estimates of the percentages of predicted x‐ and y‐coordinates falling within the 2 mm clinically acceptable threshold, along with their 95% confidence intervals, are presented in Table 2. Overall, females demonstrated greater predictive accuracy than males (65% vs. 49%). Accuracy was highest at the 1‐year prediction interval (66%), followed by the 2‐year (58%) and 4‐year (46%) intervals, indicating a progressive decline in predictive performance over longer time spans. Among the anatomical landmarks, Condylion was the most predictable (71%), followed by Point B (63%) and Gonion (61%). Menton was the least predictable landmark, with only 40% of predictions within the 2 mm threshold. Predictive accuracy was comparable between the x‐ and y‐dimensions (58% and 56%, respectively).

**TABLE 2:** Model‐based estimates of percentage of *x* and *y* coordinate predictions within clinically acceptable threshold of 2 mm.

Effect	Level	% within 2 mm (95% CI)
Dimension	*x*	58% (53%–63%)
Dimension	*y*	56% (51%–61%)
Landmark	Condylion	71% (64%–78%)
Landmark	Gonion	61% (55%–67%)
Landmark	Menton	40% (33%–47%)
Landmark	Pogonion	48% (41%–55%)
Landmark	Point B	63% (58%–68%)
Prediction interval	Prediction interval 1	66% (61%–71%)
Prediction interval	Prediction interval 2	58% (53%–64%)
Prediction interval	Prediction interval 4	46% (38%–54%)
Prediction age	Prediction age 11	53% (40%–66%)
Prediction age	Prediction age 12	66% (57%–74%)
Prediction age	Prediction age 13	67% (58%–75%)
Prediction age	Prediction age 14	49% (44%–55%)
Prediction age	Prediction age 15	62% (55%–69%)
Prediction age	Prediction age 16	52% (45%–59%)
Prediction age	Prediction age 17	46% (38%–55%)
Prediction age	Prediction age 18	59% (44%–72%)
Sex	F	65% (58%–71%)
Sex	M	49% (44%–54%)
Dimension*Landmark	x:Condylion	78% (69%–85%)
Dimension*Landmark	x:Gonion	56% (49%–63%)
Dimension*Landmark	x:Menton	38% (31%–46%)
Dimension*Landmark	x:Pogonion	48% (39%–57%)
Dimension*Landmark	x:Point B	66% (59%–73%)
Dimension*Landmark	y:Condylion	64% (55%–71%)
Dimension*Landmark	y:Gonion	67% (59%–74%)
Dimension*Landmark	y:Menton	41% (34%–49%)
Dimension*Landmark	y:Pogonion	48% (41%–55%)
Dimension*Landmark	y:Point B	59% (53%–65%)
Dimension*Prediction age	x:Prediction age 11	54% (37%–71%)
Dimension*Prediction age	x:Prediction age 12	64% (54%–73%)
Dimension*Prediction age	x:Prediction age 13	61% (51%–71%)
Dimension*Prediction age	x:Prediction age 14	50% (41%–58%)
Dimension*Prediction age	x:Prediction age 15	67% (58%–75%)
Dimension*Prediction age	x:Prediction age 16	54% (43%–64%)
Dimension*Prediction age	x:Prediction age 17	45% (31%–59%)
Dimension*Prediction age	x:Prediction age 18	68% (53%–80%)
Dimension*Prediction age	y:Prediction age 11	52% (39%–65%)
Dimension*Prediction age	y:Prediction age 12	68% (57%–77%)
Dimension*Prediction age	y:Prediction age 13	72% (63%–79%)
Dimension*Prediction age	y:Prediction age 14	49% (42%–57%)
Dimension*Prediction age	y:Prediction age 15	57% (47%–66%)
Dimension*Prediction age	y:Prediction age 16	51% (43%–59%)
Dimension*Prediction age	y:Prediction age 17	48% (42%–55%)
Dimension*Prediction age	y:Prediction age 18	49% (34%–64%)
Dimension*Sex	x:F	62% (55%–69%)
Dimension*Sex	x:M	54% (48%–59%)
Dimension*Sex	y:F	67% (61%–74%)
Dimension*Sex	y:M	44% (39%–49%)
Landmark*Prediction interval	Condylion:Prediction interval 1	72% (65%–78%)
Landmark*Prediction interval	Condylion:Prediction interval 2	70% (62%–78%)
Landmark*Prediction interval	Condylion:Prediction interval 4	72% (61%–81%)
Landmark*Prediction interval	Gonion:Prediction interval 1	71% (65%–76%)
Landmark*Prediction interval	Gonion:Prediction interval 2	65% (56%–72%)
Landmark*Prediction interval	Gonion:Prediction interval 4	48% (37%–58%)
Landmark*Prediction interval	Menton:Prediction interval 1	50% (40%–59%)
Landmark*Prediction interval	Menton:Prediction interval 2	46% (37%–55%)
Landmark*Prediction interval	Menton:Prediction interval 4	26% (18%–36%)
Landmark*Prediction interval	Pogonion:Prediction interval 1	65% (57%–73%)
Landmark*Prediction interval	Pogonion:Prediction interval 2	50% (41%–58%)
Landmark*Prediction interval	Pogonion:Prediction interval 4	30% (20%–41%)
Landmark*Prediction interval	Point B:Prediction interval 1	71% (65%–77%)
Landmark*Prediction interval	Point B:Prediction interval 2	60% (53%–67%)
Landmark*Prediction interval	Point B:Prediction interval 4	56% (47%–65%)
Landmark*Sex	Condylion:F	73% (63%–81%)
Landmark*Sex	Condylion:M	70% (61%–78%)
Landmark*Sex	Gonion:F	67% (57%–75%)
Landmark*Sex	Gonion:M	56% (50%–63%)
Landmark*Sex	Menton:F	61% (51%–71%)
Landmark*Sex	Menton:M	22% (16%–28%)
Landmark*Sex	Pogonion:F	63% (53%–72%)
Landmark*Sex	Pogonion:M	33% (25%–42%)
Landmark*Sex	Point B:F	60% (52%–68%)
Landmark*Sex	Point B:M	65% (59%–71%)

Model‐based estimates of the percentages of Euclidean distances between actual and predicted landmark positions within the 2 mm clinically acceptable threshold, together with 95% confidence intervals, are presented in Table 3. Overall predictive accuracy was lower when assessed using Euclidean distance compared with separate coordinate analysis. Females again demonstrated greater predictability than males (35% vs. 22%). The highest accuracy was observed at the 1‐year interval (39%), followed by the 2‐year (28%) and 4‐year (20%) intervals. Condylion remained the most predictable landmark (39%), followed by Gonion (32%) and Point B (31%), whereas Menton was again the least predictable (18%).

**TABLE 3:** Model‐based estimates of percentage of Euclidian distances within clinically acceptable threshold of 2 mm.

Effect	Level	% within 2 mm (95% CI)
Landmark	Condylion	39% (30%–49%)
Landmark	Gonion	32% (25%–40%)
Landmark	Menton	18% (13%–24%)
Landmark	Pogonion	24% (18%–30%)
Landmark	Point B	31% (26%–37%)
Prediction interval	Prediction interval 1	39% (33%–44%)
Prediction interval	Prediction interval 2	28% (23%–35%)
Prediction interval	Prediction interval 4	20% (14%–26%)
Prediction age	Prediction age 11	27% (16%–42%)
Prediction age	Prediction age 12	34% (24%–45%)
Prediction age	Prediction age 13	37% (28%–47%)
Prediction age	Prediction age 14	20% (15%–27%)
Prediction age	Prediction age 15	34% (24%–46%)
Prediction age	Prediction age 16	27% (21%–35%)
Prediction age	Prediction age 17	15% (10%–22%)
Prediction age	Prediction age 18	37% (22%–55%)
Sex	F	35% (28%–43%)
Sex	M	22% (18%–27%)
Landmark*Sex	Condylion:F	41% (28%–56%)
Landmark*Sex	Condylion:M	37% (27%–49%)
Landmark*Sex	Gonion:F	34% (24%–46%)
Landmark*Sex	Gonion:M	30% (23%–38%)
Landmark*Sex	Menton:F	40% (30%–52%)
Landmark*Sex	Menton:M	7% (4%–11%)
Landmark*Sex	Pogonion:F	33% (24%–43%)
Landmark*Sex	Pogonion:M	16% (11%–23%)
Landmark*Sex	Point B:F	28% (20%–38%)
Landmark*Sex	Point B:M	35% (28%–42%)

## 4 Discussion

Accurate prediction of craniofacial growth remains a major challenge in orthodontics, particularly during puberty when mandibular development is highly variable. Reliable prediction of mandibular landmark displacement can improve treatment timing and long‐term stability [30]. This study evaluated AR modelling for longitudinal prediction of mandibular landmarks using clinically relevant accuracy thresholds, providing quantitative insight into when and where growth prediction is most reliable.

We selected an AR‐style statistical model with ridge‐regularised linear regression because the amount of usable supervised data in AAOF was limited for our task. Although the dataset contains many cephalograms, only a subset includes complete annotated $\left(x,y\right)$ landmark coordinates. As a result, the effective number of labelled input–output pairs were relatively small compared to what is typically needed to train deep neural networks reliably. In this regime, high‐capacity models can easily overfit to annotation noise or subject‐specific patterns, making their generalisation difficult to trust. In contrast, an AR formulation is simple to understand and align with the clinical expectation that landmark locations change gradually between consecutive visits. Implementing the model as a ridge‐regularised linear regressor keeps the number of trainable parameters small, and the learned update rule easy to interpret, which makes the model outputs straightforward to inspect and explain. This transparency is valuable in cephalometric applications because we can directly reason about the predicted landmark movement from one visit to the next, rather than relying on a black‐box mapping.

Our results showed a significant difference between prediction accuracy evaluated separately for x‐ and y‐coordinates and accuracy assessed using two‐dimensional Euclidean distance. While coordinate‐based analyses demonstrated moderate predictive performance, Euclidean distance–based accuracy was substantially lower, likely because small independent errors along each axis accumulate when combined into a single two‐dimensional displacement measure. From a clinical perspective, Euclidean distance may more closely reflect the true accuracy of landmark position prediction, as craniofacial growth and landmark displacement occur simultaneously in both spatial dimensions. Therefore, coordinate‐based accuracy values may overestimate the practical clinical performance of the model if interpreted in isolation. These findings highlight the importance of incorporating multidimensional evaluation metrics when assessing predictive models intended for clinical craniofacial applications.

In terms of sex comparison, females demonstrated greater predictive accuracy than males across both coordinate‐based and Euclidean distance analyses. This likely reflects sex differences in growth timing and variability, as female craniofacial growth typically occurs earlier and over a shorter, more stable period, whereas male growth is more prolonged and variable [31, 32]. Increased biological variability in males may therefore reduce predictability rather than reflect model limitations, consistent with findings from previous longitudinal growth studies [32, 33].

Prediction age demonstrated only a marginally significant effect on predictive accuracy when evaluated using Euclidean distance, whereas no significant effect of prediction age was observed when the *x*‐ and *y*‐coordinates were analysed separately, suggesting that overall spatial displacement may better capture subtle cumulative growth‐related changes. This observation is in accordance with the literature indicating that skeletal maturation status and growth velocity are more informative indicators of craniofacial development than chronological age alone [34, 35]. Chronological age often fails to reflect individual variation in biological growth timing, particularly during periods of accelerated developmental change. Furthermore, because AR models primarily rely on recent observations to forecast future values, predictions are inherently influenced more by the immediate growth trajectory than by absolute age [36]. Accordingly, the present findings suggest that short‐term growth dynamics rather than chronological age play a greater role in determining prediction accuracy, consistent with established concepts of individualised and phase‐dependent craniofacial growth patterns described in longitudinal growth studies [37, 38].

The AR models were able to predict mandibular landmark displacement within the 2 mm clinically acceptable threshold with moderate accuracy, particularly for shorter prediction intervals. The highest predictive accuracy was consistently observed at the 1‐year interval, with performance progressively decreasing at 2‐ and 4‐year intervals. This indicates that AR modelling is more reliable for short‐term growth prediction than for longer‐term forecasting. This decline likely reflects increasing biological nonlinearity over time, including variability in pubertal growth timing and magnitude. Similar reductions in accuracy with longer follow‐up timepoints have been reported in longitudinal craniofacial prediction studies [38].

Landmark type had a significant main effect on predictive accuracy. Condylion was consistently the most predictable landmark, whereas Menton was the least predictable. This pattern agrees with recent studies suggesting that condylar growth follows relatively consistent modelling pathways, while Menton displacement is influenced by vertical growth, mandibular rotation, and dentoalveolar adaptation, increasing positional variability over time [39, 40]. In addition, Menton represents the most inferior point on the mandibular symphysis and is therefore particularly sensitive to cumulative rotational and translational changes occurring throughout craniofacial growth. Small variations in mandibular plane rotation, lower anterior facial height development, dental eruption, and alveolar compensation may substantially alter Menton position in both vertical and anteroposterior dimensions. These complex and interacting developmental processes may not be adequately captured by an AR model relying primarily on prior landmark position. Clinically, the lower predictive accuracy observed for Menton suggests that caution is warranted when interpreting model predictions for mandibular vertical development or treatment decisions that depend heavily on accurate prediction of chin position and mandibular rotation. Gonion and Point B demonstrated intermediate accuracy, further supporting evidence that mandibular growth predictability is inherently landmark‐dependent and varies across anatomical regions rather than occurring uniformly throughout the mandible.

### 4.1 Clinical Implications

The clinical implications of the observed prediction accuracy should be interpreted cautiously. In this study, a 2 mm threshold was selected as a clinically meaningful cutoff because establishing clinically acceptable error thresholds is important when evaluating the translational potential of predictive models in orthodontics. Although a prediction error within 2 mm may be acceptable for assessing general growth patterns, it may still be insufficient for precise clinical decision‐making in cases requiring high‐stakes decisions such as extraction/non‐extraction or surgery/non‐surgery. Even under the best‐performing conditions in the present study, approximately 30% of predictions exceeded this threshold, indicating that the current model should be considered primarily as a proof‐of‐concept rather than a clinically deployable tool. The lower overall predictive accuracy observed in male subjects, in which approximately half of predictions exceeded the 2 mm threshold, further highlights the challenges of modeling mandibular growth during periods of greater biologic variability and prolonged pubertal growth. These findings suggest that reliance on prior landmark position alone may be insufficient to accurately capture growth dynamics in male patients. Incorporation of additional biologically relevant variables, such as skeletal maturity indicators, pubertal growth stage, and individual growth velocity measures, may improve prediction performance by better accounting for interindividual variability in craniofacial growth and development.

### 4.2 Limitations

Several limitations should be acknowledged in the present study. To differentiate physiologic growth‐related changes from treatment‐induced changes in mandibular morphology and landmark displacement, orthodontically treated cases were excluded from the analysis. However, residual heterogeneity inherent to retrospective longitudinal datasets may still have influenced the learned model parameters and should be considered when interpreting the findings. In addition, sparse data within certain factor combinations limited full factorial modelling and may have reduced the ability to detect higher‐order interactions. The AR model relied primarily on chronological age and prior landmark position without incorporation of skeletal maturity indicators such as cervical vertebral maturation stages, pubertal growth status, or individual growth velocity, all of which may substantially influence mandibular development. Because the timing, magnitude, and direction of mandibular growth are closely associated with biologic maturation rather than chronological age alone, subjects of the same age may exhibit substantially different craniofacial growth trajectories depending on their pubertal growth status. The absence of skeletal maturity information may therefore have contributed to increased variability in landmark displacement patterns and reduced predictive accuracy, particularly during periods of rapid adolescent growth. Furthermore, prediction accuracy was evaluated using a fixed 2‐mm threshold as a clinically meaningful cutoff; however, the clinical significance of this threshold may vary depending on the anatomical landmark and specific clinical application. Additional limitations include the relatively small sample size and the inherent variability associated with manual cephalometric landmark identification, both of which may have affected model performance. Despite these limitations, the results are encouraging and support the feasibility of modelling craniofacial growth trajectories as a time‐series prediction problem.

### 4.3 Future Directions for Research

Future studies should incorporate larger and more balanced longitudinal datasets to improve prediction performance and model robustness. In addition, incorporation of skeletal maturation indicators rather than reliance on chronological age alone may improve characterisation of individual growth phases and enhance the biologic relevance, predictive accuracy, and clinical applicability of craniofacial growth prediction algorithms. Because mandibular growth patterns may vary across Angle classifications, future investigations should also evaluate model performance separately in Class I, Class II, and Class III subjects to determine whether prediction accuracy differs among clinically relevant malocclusion groups. In addition, because mandibular displacement patterns may differ according to facial growth type, future studies should investigate whether incorporation of vertical, average, and horizontal growth pattern classifications improves prediction accuracy, particularly for landmarks such as Menton that are strongly influenced by mandibular rotation and vertical facial development. Additional improvements may be achieved through automated landmark detection methods that reduce annotation variability and through the application of more advanced nonlinear and deep learning–based temporal prediction algorithms capable of capturing complex craniofacial growth dynamics and growth phase transitions more effectively. Furthermore, because the model was developed and evaluated exclusively using the AAOF Legacy Collection, its generalisability to populations with different racial backgrounds, geographic regions, or clinical settings remains uncertain. Therefore, external validation using independent datasets will be necessary to confirm model robustness and broader clinical applicability.

## 5 Conclusions

Based on the findings of this study, AR models demonstrate clinically acceptable short‐term predictive capability for mandibular landmark displacement during puberty; however, predictive accuracy declines as the prediction interval increases. Predictive performance was significantly influenced by landmark type and sex. Females consistently demonstrated greater predictive accuracy than males in both coordinate‐based and Euclidean distance analyses. Furthermore, prediction accuracy varied by anatomical location, with certain landmarks—particularly Condylion—showing higher predictability than others. Evaluation using multidimensional (Euclidean) distance provided a more stringent assessment of model performance and resulted in lower apparent accuracy compared with separate *x*‐ and *y*‐coordinate analyses. While prediction accuracy was comparable between horizontal and vertical dimensions, it was clearly site‐specific.

## Author Contributions

Abdullah Al Fahad contributed to design of the study, acquisition, analysis, and interpretation of the data, and drafted and critically revised the manuscript and gave final approval. Jared Brown contributed to acquisition, analysis, and interpretation of the data, and drafted and critically revised the manuscript and gave final approval. Ali Walid Elhag contributed to acquisition of the data, critically revised the manuscript and gave final approval. Azeezjamal Samad Lakhani contributed to acquisition of the data, critically revised the manuscript and gave final approval. George Eckert contributed to analysis, and interpretation of the data, critically revised the manuscript and gave final approval. Marwa Baraka contributed to interpretation of the data, and drafted and critically revised the manuscript and gave final approval. Mohammad Al Hasan contributed to design of the study, acquisition, analysis, and interpretation of the data, and drafted and critically revised the manuscript and gave final approval. Hakan Turkkahraman contributed to design of the study, acquisition, analysis, and interpretation of the data, and drafted and critically revised the manuscript and gave final approval.

## Funding

The authors have nothing to report.

## Ethics Statement

This study was reviewed by the Institutional Review Board (IRB) and determined to be not human subjects research (NHSR); therefore, no further IRB review was required.

## Conflicts of Interest

The authors declare no conflicts of interest.

## Data Availability

The data that support the findings of this study are available from the corresponding author upon reasonable request.
